# Influence of Co-Sputtered Ag:Al Ultra-Thin Layers in Transparent V_2_O_5_/Ag:Al/AZO Hole-Selective Electrodes for Silicon Solar Cells

**DOI:** 10.3390/ma13214905

**Published:** 2020-10-31

**Authors:** Thomas Tom, Eloi Ros, Nicolau López-Pintó, José Miguel Asensi, Jordi Andreu, Joan Bertomeu, Joaquim Puigdollers, Cristobal Voz

**Affiliations:** 1 Departament de Física Aplicada, Universitat de Barcelona, 08028 Barcelona, Spain; thomastom@ub.edu (T.T.); nicolau.lopez.pi@ub.edu (N.L.-P.); jmasensi@ub.edu (J.M.A.); Jordi.andreu@ub.edu (J.A.); 2 Institute of Nanoscience and Nanotechnology (IN2UB), Universitat de Barcelona, 08028 Barcelona, Spain; 3 Departament d’Enginyeria Electrònica, Universitat Politècnica de Catalunya, 08034 Barcelona, Spain; eloi.ros@upc.edu (E.R.); joaquim.puigdollers@upc.edu (J.P.)

**Keywords:** heterojunctions, contacts, silicon, solar cells, sputtering, transparent films, transition metal oxide

## Abstract

As optoelectronic devices continue to improve, control over film thickness has become crucial, especially in applications that require ultra-thin films. A variety of undesired effects may arise depending on the specific growth mechanism of each material, for instance a percolation threshold thickness is present in Volmer-Webber growth of materials such as silver. In this paper, we explore the introduction of aluminum in silver films as a mechanism to grow ultrathin metallic films of high transparency and low sheet resistance, suitable for many optoelectronic applications. Furthermore, we implemented such ultra-thin metallic films in Dielectric/Metal/Dielectric (DMD) structures based on Aluminum-doped Zinc Oxide (AZO) as the dielectric with an ultra-thin silver aluminum (Ag:Al) metallic interlayer. The multilayer structures were deposited by magnetron sputtering, which offers an industrial advantage and superior reliability over thermally evaporated DMDs. Finally, we tested the optimized DMD structures as a front contact for n-type silicon solar cells by introducing a hole-selective vanadium pentoxide (V_2_O_5_) dielectric layer.

## 1. Introduction

The proliferation of photonic technologies seen in recent years has been enabled by a steadily increasing potential of the optoelectronic devices. Opening a new fan out of applications including sensing, anti-reflection coatings or energy harvesting devices among many others. Many of these applications employ pure metal and metal-containing films (e.g., Ag coated waveguides, V thin film optical switches etc. [1]). For those applications examples, the most important characteristics of the films are related to the film thickness, either directly dependent or indirectly. Therefore, it is very interesting to study an effective method to deposit atomically smooth ultra-thin metallic layers. Moreover, ultra-thin metallic layers are a very interesting alternative for their use in transparent conductive electrodes. Traditionally, these electrodes have been based on degenerate doped wide band gap semiconductors such as gallium-doped zinc oxide (GZO), fluorine-doped tin oxide (FTO) or tin-doped indium oxide (ITO) [2,3,4]. Transparent conducting oxides (TCOs) are widely used in research and industrial applications including LCD screens, touch panels, transparent electronics, or photovoltaic devices [5,6,7,8]. Particularly for photovoltaics, ITO is commonly used as the front transparent conductive electrode and plays a fundamental role in many types of solar cells, from conventional crystalline silicon solar cells to organic solar cells [9,10,11]. However due to its high-temperature processing, brittle nature, increasing cost due to the scarcity of indium and disadvantages in the terms of production [12,13], a continuous effort is being done in order to find suitable indium-free alternatives to ITO. Metallic nanowire networks, carbon nanotubes, conductive polymers, graphene, and ultra-thin metallic films [14,15,16,17,18] are some of the alternatives to potentially reduce the production cost in the photovoltaic (PV) industry. Recently, dielectric-metal-dielectric (DMD) structures have emerged as valid candidates to substitute the ITO electrode in silicon solar cells. DMD structures based on V_2_O_5_/Ag/V_2_O_5_, AZO/Ag/TiO_2_ have been previously studied and were fabricated using the thermal evaporation technique [19,20]. The main disadvantage of this method is the agglomeration of the metal, related to the particular growth mode (i.e., Volmer-Webber type) of the deposited film. Therefore, it becomes a necessity to deposit a seed (wetting) layer to avoid migration of metal atoms over the surface to form clusters [21,22]. The advantage of using a gold seed layer to avoid agglomeration in dopant-free Si solar cells using V_2_O_5_/Ag/V_2_O_5_ as selective contacts was demonstrated in [23]. Nevertheless, the deposition of a few nanometers gold seed layer by thermal evaporation could not easily scale up to industrial production.

Besides the great importance of the metallic layer in DMDs, choosing the proper dielectric is crucial for the final optoelectronic properties of the multi-layered stack. In this sense, transition metal oxides (TMOs) (e.g., vanadium, tungsten, and molybdenum oxides) are very interesting dielectrics for DMD structures. Some of the interesting features of TMOs are their refractive index, their large work function that ranges from 2 to 7 eV and their wide bandgap of more than 3 eV [24]. These materials possess an extraordinary transparency to visible light and can be used as the anti-reflective coating (ARC) on silicon due to their bandgap and refractive index. Furthermore, the deep work function allows TMOs to actively participate in the band structure of the device, selectively collecting holes over electrons.

Selective conductivity of holes and electrons is used to induce junctions or provide ohmic contacts with semiconductor materials (e.g., perovskite, c-Si or organic). Exploring and developing new hole and electron selective contacts has motivated increasingly active research in recent years. Particularly for crystalline silicon solar cells the interest has been due to the simple fabrication process and the rather high efficiencies obtained. Conventional crystalline silicon (c-Si) solar cells using the doped amorphous silicon (a-Si: H) as p and n layers have achieved a high record power conversion efficiency (PCE) of more than 25% [25,26]. Nevertheless, by choosing the appropriate materials for the charge transport layers (CTL) one can accomplish selective transport of one carrier while simultaneously blocking the other without the need for doping. Transition metal oxides have demonstrated excellent carrier selective properties as they work as hole-selective contacts for heterojunction silicon solar cells [27]. Among these, MoO_3_ as a hole-selective contact has obtained the highest PCE of more than 22% [28]. Other p-type materials such as graphene, CNTs and MXene also have shown an excellent hole-selective nature [29,30,31]. However, dopant-free heterojunction solar cells still need transparent conducting oxides to improve the series resistance of the device and the short-circuit current density (J_sc_).

In this work the magnetron radiofrequency (RF) sputtering technique was used as a replacement of thermal evaporation to deposit DMDs. RF sputtering is an industrially suitable technique with excellent uniformity on large areas, high adhesion, purity, and high deposition rates [32,33,34]. Moreover, sputtering enables better control over thickness of the metallic layers [35,36] and may eliminate the more complex usage of a seed layer [37].

First, we developed a completely sputtered, cost-effective and stable DMD layer of AZO/Ag:Al/AZO and investigated its electrical and optical characteristics. To avoid clustering of the metal nanostructures in the ultra-thin films the Ag was co-sputtered with Al [38]. However, the AZO/Ag:Al/AZO layer does not promote a preferred hole-selectiveness. Later, it was accomplished by replacing the bottom AZO layer with a high-quality hole-selective V_2_O_5_ layer. Silicon solar cells were then fabricated using a front V_2_O_5_/Ag:Al/AZO multilayer electrode, which was optimized for the thickness of the V_2_O_5_.

## 2. Materials and Methods

For examining and optimizing the properties of the DMD AZO/Ag:Al/AZO multilayers these structures were deposited on Corning glass 1737F. Prior to deposition the substrate was cleaned using mild soap, deionized water and isopropanol and then dried using N_2_ gas. For the fabrication of solar cells, silicon substrate (n-type) was treated with hydrofluoric (HF) acid (1%) for 1 min before the deposition. All thin films were deposited by means of rf sputtering (ATC ORION 8 HV) using a 3-inch target. The vacuum chamber was pumped to a base pressure of 3 × 10^−6^ Torr prior to all the depositions. AZO films were deposited from ZnO:Al target (2 wt% of Al) of 99.99% purity. The depositions were done at different pressures in the range between 1 and 8 mTorr with a constant power of 150 W. Reactive argon hydrogen (Ar:H_2_) atmosphere (3.5% of H_2_) was maintained in the vacuum chamber. The films were deposited at room temperature and at 100 °C with a deposition rate of 3.5 nm/min. The ultra-thin sub-nanometer roughness Ag:Al metallic films were prepared by co-sputtering of Al and Ag at room temperature. The sputtering power of the Ag target (99.99% pure) was kept constant at 100 W whereas the power applied to Al target (99.99% pure) varied from 100 W to 175 W. The sputtering was performed in an atmosphere of argon and all the depositions were done at a base pressure of 1 mTorr. Thickness of the films was estimated from the deposition rates of 100 nm thick films of Al and Ag on glass at similar conditions. Thus, the Al content in the Ag:Al films can be estimated about 16% to 20% in different samples.

**Figure 1:**
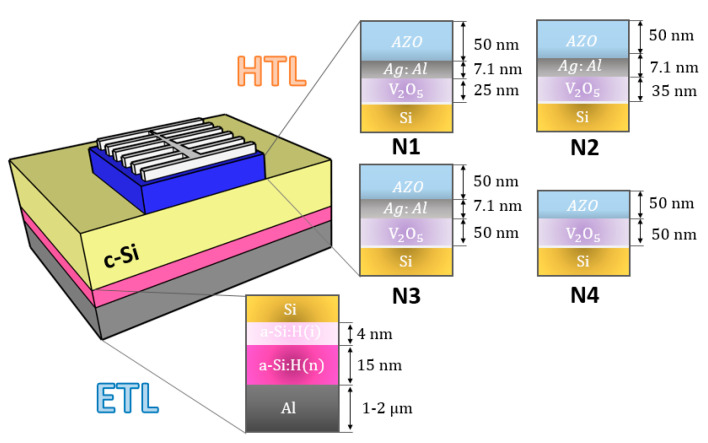
Schematic of a solar cell based on n-type c-Si, with different configurations studied in this work. The multi structured layers of V_2_O_5_/Ag:Al/AZO act as both transparent conducting and hole-selective electrode.

The DMD structures (AZO/Ag:Al/AZO) investigated in this work were fabricated by sandwiching ultra-thin Al:Ag films between two AZO layers. All the layers were grown by sputtering and thickness of the films was measured using both an Alpha-Step D-120 profilometer (Scientec, Les Ulis, France) and a SD 2100 ellipsometre (Plasmos, Munich, Germany). Sheet resistance (R_sh_) of the films was measured using a four-point probe set-up (Jandel, Grand Union House, Leighton Buzzard UK). UV-Visible-NIR spectrophotometer Lambda 950 (Perkin Elmer, Shelton, CT, USA) was used to measure the optical transmittance of the films. The surface morphology and the percolation in the films were investigated by atomic force microscope (AFM) (Bruker Multimode 8 with Nanoscope, Santa Barbara, CA, USA), and SEM images (JEOL JSM-7001F, Tokyo, Japan). N-type (100) c-Si wafers with resistivity of 2 Ωcm, thickness of 280 µm, one-side polished, non-textured were used for the fabrication of solar cells. Four different solar cell architectures were developed with modifications in the hole-selective layer. One without sandwiching Ag:Al layer in AZO and V_2_O_5_, other three by incorporating Ag:Al layer with varied thickness of V_2_O_5_. Thermally evaporated V_2_O_5_ was used as the dopant-free hole-selective contact as reported in our previous work [23]. An intrinsic (i-type) a-Si:H and doped n-type a-Si:H layers were deposited on the rear side by plasma-enhanced chemical vapor deposition (PECVD) to provide a reference electron selective contact [39]. The lithographic patterning of 1 cm^2^ was defined as the active area and a 2-µm-thick Ag grid (4.5% shadow) was thermally evaporated as the front contact. Finally, the back-contact metallization was accomplished by thermally evaporating Al (1µm thick). A scheme of the prior explained device structure with varying parameters in the hole-selective layer are schematically described in Figure 1. Current density-voltage (J-V) characteristics of the fabricated solar cells were measured using a 94041A solar simulator (Newport, Irvine, CA, USA). External quantum efficiency (EQE) curves were measured using a QEX10 set-up (PV Measurements, Point Roberts, WA, USA).

## 3. Results and Discussions

Electrical and optical characterization of the sputtered AZO films was carried out. The total thickness of all the films was kept around 75 nm that provides a good antireflection property [40,41].

**Figure 2:**
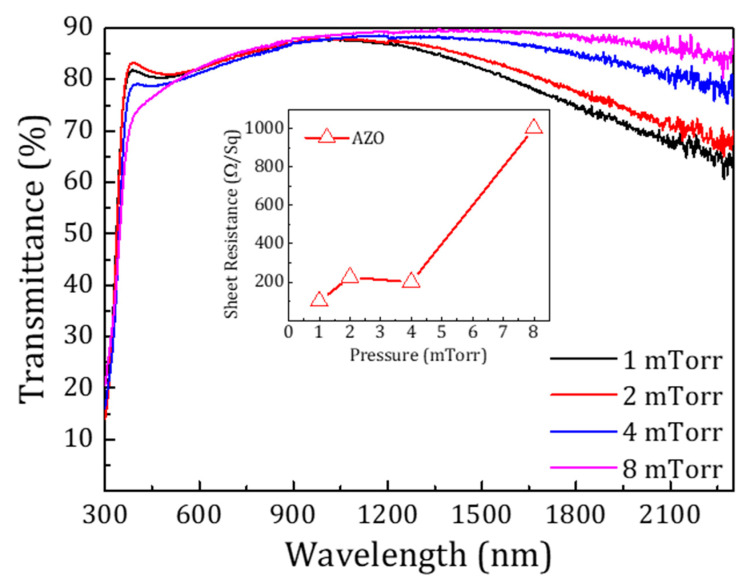
Optical transmittance of AZO films sputtered in Ar:H_2_ atmosphere at varying pressure. Overall transmittance in the visible region was independent of the pressure. The inset shows the decrease in the sheet resistance of the films at lower pressures.

Films were studied with varying pressure, keeping the temperature (100 °C) and power (150 W) as constant deposition parameters. A huge decrease in sheet resistance from kΩ/Sq to around 100 Ω/Sq was observed in the films by reducing the deposition pressure from 8 to 1 mTorr. This effect could be explained by the formation of oxygen vacancies or a higher hydrogen reduction of the films. This agrees with the steady decrease in the infrared transmittance of the films (see Figure 2) as the deposition pressure is reduced [42,43]. Transmittance measurements for the AZO films indicate that all the films grown at different pressures show an average transmittance of 80%. The transmittance of the films in the visible range was independent of the pressure as shown in Figure 2, whereas the inset shows the drop in sheet resistance as a function of the deposition pressure.

**Figure 3:**
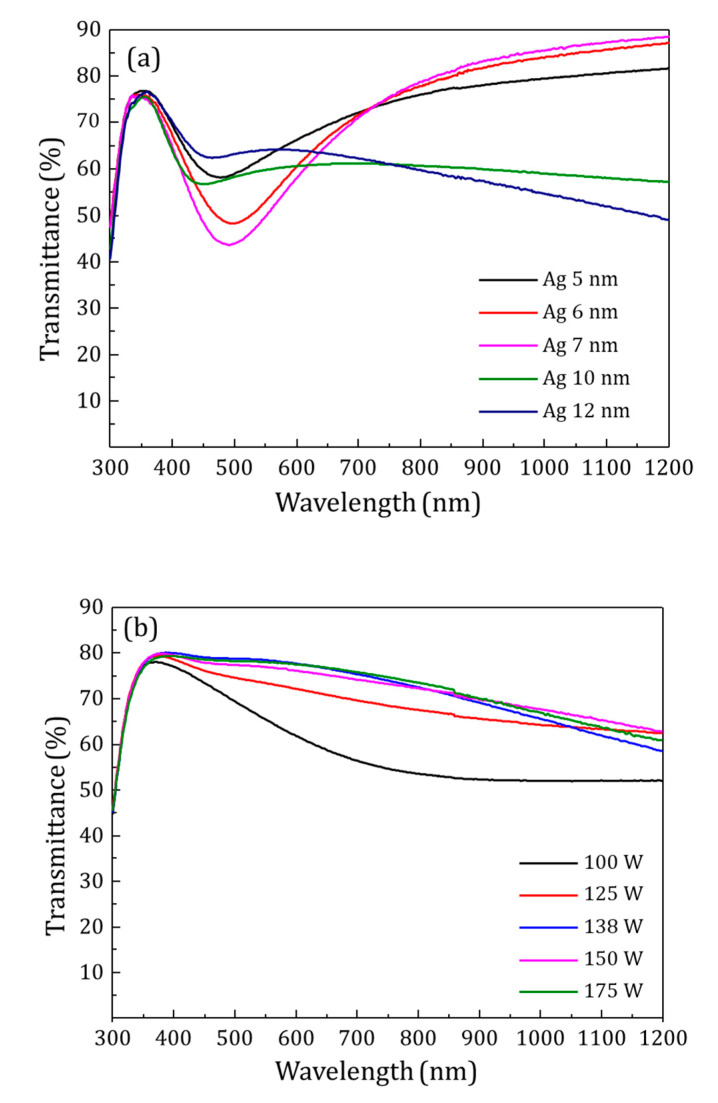
(**a**). Optical transmittance of Ag layers with varying thicknesses deposited on glass. The broad dip in transmittance were observed due to the discontinuity in the films. (**b**). Optical transmittance of Ag:Al films deposited on glass. Optimizing the doping of Al with different sputtering powers on Al target, fixing the Ag power at 100 W.

To improve the electric properties of the AZO films, an ultra-thin Ag film was sandwiched between two AZO layers. The optical properties and sheet resistance of this intermediate ultra-thin Ag films for thickness ranging from 5 to 12 nm were studied. Results showed that the broad drop in transmittance spectra within the visible region could be explained by the Volmer-Weber growth model [44], i.e., the tendency of silver nanoparticles to form islands during the first few nanometers (see Figure 3a). The absorption caused by the formation of localized surface plasmons would explain the comparatively low optical transmittance and electrical discontinuity [45,46]. Therefore, the pure Ag films were highly resistive, reaching sheet resistances greater than 10^5^ Ω/Sq until a threshold thickness of 10 nm that leads to a sheet resistance of 2 × 10^3^ Ω/Sq.

To obtain ultra-smooth Ag-based films with reduced RMS roughness, electrical continuity and higher transmittance, Ag:Al films were prepared by co-sputtering of Al with Ag. Al doping enhances the density of heterogeneous nucleation sites, thus larger nuclear density and smaller particle size [38]. First, Ag:Al films were optimized for Al doping concentration with a constant power on Ag target. Figure 3b shows the transmittance of films deposited at varying powers of Al from 100 W to 175 W keeping constant the power of Ag 100 W. The higher transmittance and lower sheet resistance were obtained for the Ag:Al films sputtered at 138 W, with a transmittance of 75–80% in the visible region and a sheet resistance of 60 Ω/Sq. All other films showed higher sheet resistance.

**Figure 4:**
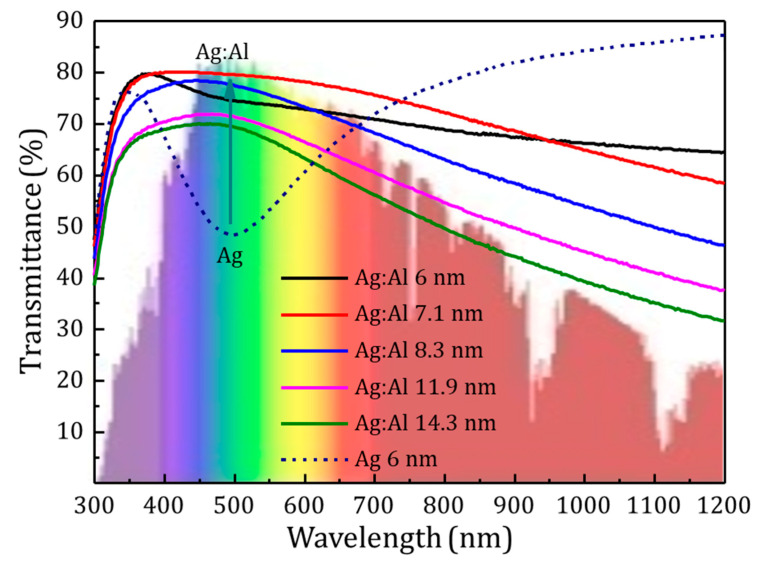
Optical transmittance of Ag:Al samples deposited on glass. A sample with 6 nm thick Ag layer without Al doping is also shown for comparison. A 30% increase in the transmittance was observed due to the doping.

Then several Ag:Al films with varying thickness co-sputtered with Al at 138 W have been studied. Figure 4 shows the comparison of optical transmittance of the pure Ag film of 6 nm layer and Ag:Al films with thickness ranging from 6 nm to 14 nm.

**Figure 5:**
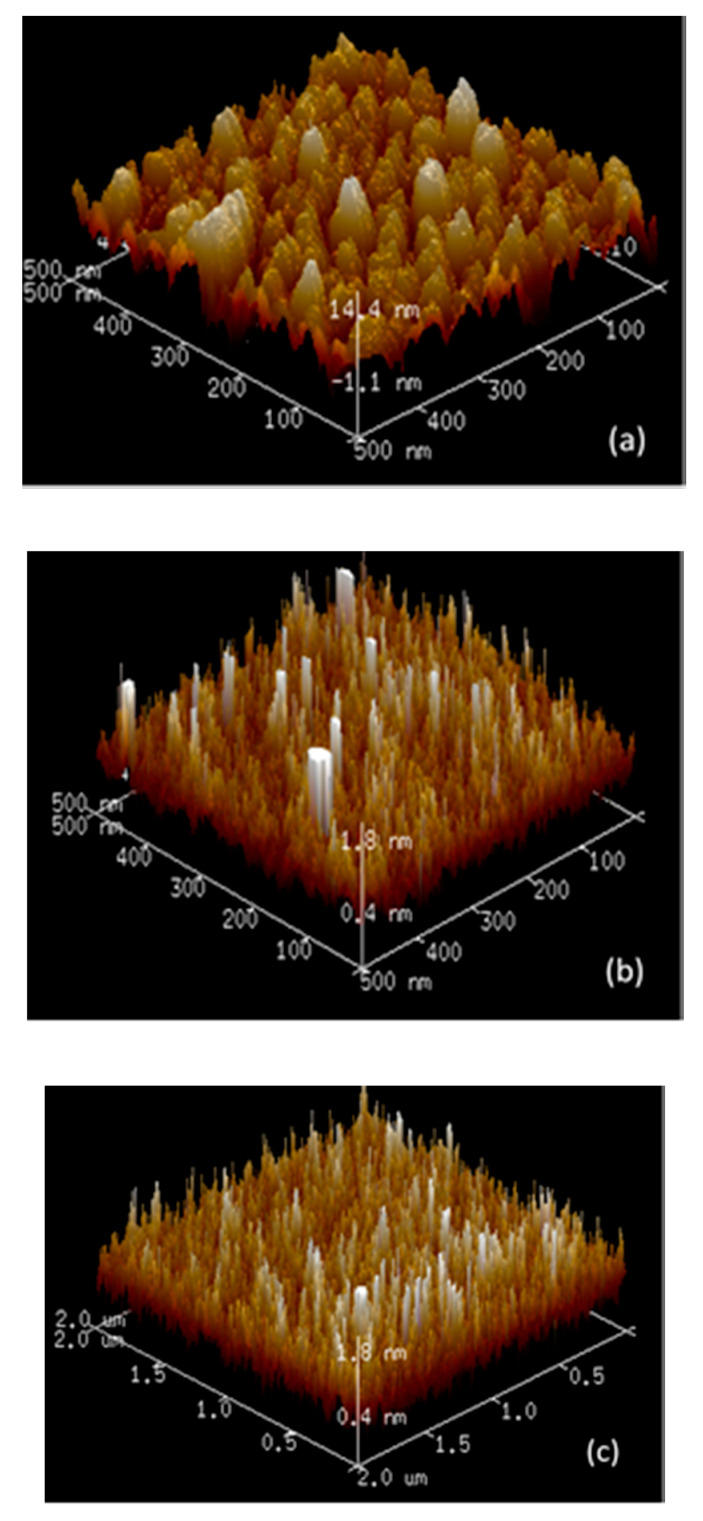
AFM images of (**a**) 6 nm Ag film with an RMS roughness of 3.99 nm, (**b**) 8.3 nm Ag:Al films with an RMS roughness of 0.47 nm, (**c**) 7.1 nm Ag:Al films with an RMS roughness of 0.37 nm.

**Figure 6:**
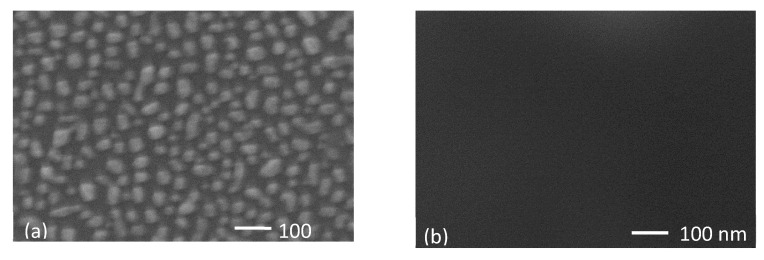
Comparison of SEM micrographs (**a**) Ag film of 6 nm, (**b**) Ag:Al film of 7.1 nm films. Al doping reduces the clustering and aids to form more continuous films.

For the same thickness of Ag and Ag:Al samples, an increase of 30% transmittance was obtained for the later in the visible region. Thus, aiding to compensate for the broad dip in the transmittance spectra in ultra-thin Ag films. The atomic force microscope (AFM) images of the surface morphology of a 7 nm pure Ag film and 8.3 nm and 7.1 nm Ag:Al films are shown in Figure 5. The peak-to-valley height of pure Ag films with a maximum value of 14.4 nm and Ag:Al films with 1.8 nm exhibits the smoothness of the films. The RMS roughness value obtained for pure Ag film of 7 nm was 3.99 nm and for Ag:Al films with thicknesses 8.3 nm and 7.1 nm were 0.47 nm and 0.37 nm respectively. This lower RMS value confirms the ultra-smooth nature of the films. Figure 6 shows the SEM micrographs of the above-mentioned samples, which confirms the continuity in the films. The clustering of pure Ag films and the homogeneity in the Ag:Al films were easily observable.

Grazing incidence X-ray diffraction analysis evidenced some crystalline microstructure for the Ag:Al film observed in Figure 6b, which is quite remarkable for such an ultrathin film (i.e., 7.1 nm). On the other hand, the V_2_O_5_ layer was completely amorphous and the AZO layer evidenced an oriented pattern compatible with ZnO-Hexagonal phase.

**Figure 7:**
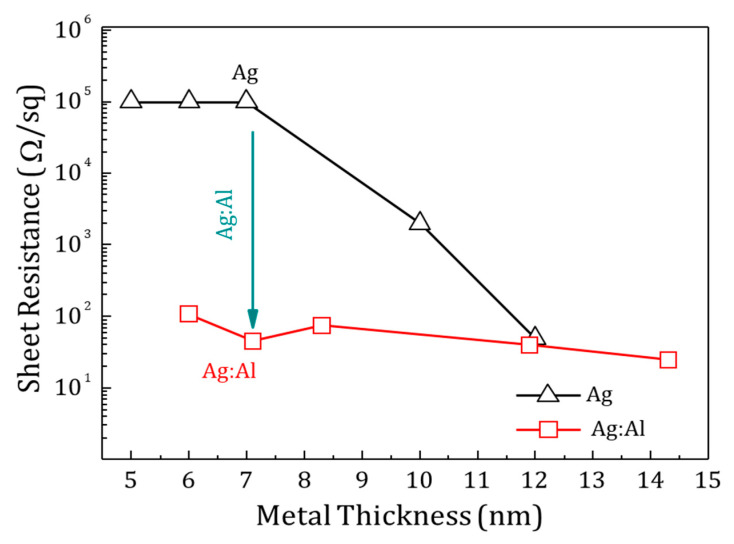
The sheet resistance as a function of thickness for Ag and Ag:Al films are shown. The doping reduces the formation of isolated islands and hence the sheet resistance. The inset compares the resistivity of the Ag and Ag:Al films as a function of thickness.

Sheet resistance with the increasing thickness of Ag and Ag:Al films are shown in Figure 7. The Ag films with thickness less than 7 nm behave as insulators with a high sheet resistance of 10^5^ Ω/Sq. Ag:Al films of similar thickness show a sheet resistance of several 100 Ω/Sq. The huge drop in sheet resistance in Ag:Al films was due to the enhanced density of heterogeneous nucleation sites, avoiding the formation of isolated islands in the films [45]. The calculated resistivity of the Ag:Al films was comparable to the bulk resistivity of Ag films, confirming the uniform nature of the films.

**Figure 8:**
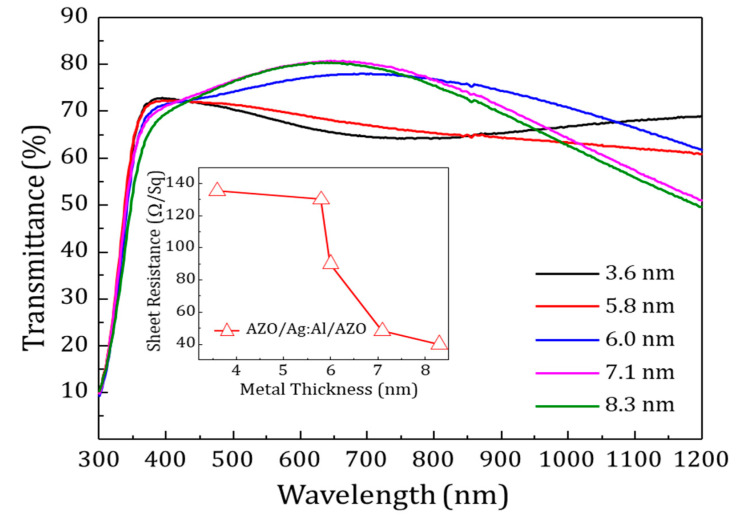
Optical transmittance of AZO/Ag:Al/AZO (18 nm/Ag:Al/70 nm) multilayer structure deposited on glass is shown. Inset shows the sheet resistance as a function of metal thickness. Optical combination of high transparency and lower sheet resistance was obtained for the metal thickness of 7.1 nm.

The influence of the Ag:Al layers on the AZO/Ag:Al/AZO structures was investigated by studying the electrical and optical properties of the multilayers. Figure 8 shows the transmittance spectra for various metal thicknesses and the inset displays the sheet resistance as a function of metal thickness for AZO/Ag:Al/AZO multilayer.

The thickness of the AZO layers was fixed as 18 nm and 70 nm for the bottom and top layer respectively. The optimized Ag:Al films sputtered at 138 W with varying thickness from 3 to 8.1 nm were used for the study. As the thickness of the intermediate layer increased, a decrease in sheet resistance and an increase in transmittance was observed in the multilayer structure. The intermediate layer of 7.1 nm showed the best result with a lower sheet resistance of 48 Ω/Sq and higher transmittance of 80% in the visible region.

Considering the final step to fabricate TCO for devices, figure of merit (FOM) values were calculated for these structures. The FOM defined by Haacke, Ф_TC_ = T^10^/R_s_ [47], was calculated in the range 400–800 nm for ITO, AZO thin film and multi-layered structure, and the values are given in Table 1. The FOMs were estimated for reduced AZO films (Ar:H_2_ atmosphere) at 100 °C and at room temperature (RT) and for non-reduced AZO films (Ar atmosphere) at RT. The multilayer structure attained the highest FOM of 1.9 × 10^−3^ Ω^−1^ compared to other films, which layed between 2 × 10^−5^ Ω^−1^ and 1.1 × 10^−3^ Ω^−1^. The FOM values achieved here are comparable to state of art TCOs used as antireflection coating in Si heterojunction solar cells, as reported by M.Nisha et al. [4]. This shows the potential of AZO/Ag:Al/AZO multilayer to replace ITO, which could be used as transparent conducting film in various optoelectronic devices such as large-area solar cells, diodes, photodetectors etc.

**Figure 9:**
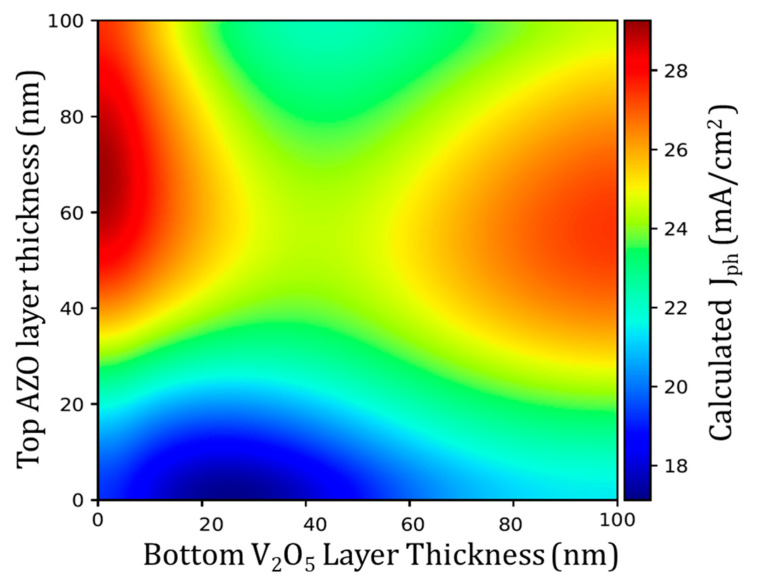
Mapping of the photogenerated current J_ph_ under AM 1.5 irradiance as function of top AZO and bottom V_2_O_5_ thicknesses. The Ag:Al intermediate layer thickness was fixed at 7.1 nm. AZO layer between 50 and 60 nm thickness and V_2_O_5_ layer between 20 and 50 nm thickness shows better optical performance.

A carrier selective layer must be included to implement this electrode in photovoltaic devices. Thus, later studies were conducted to replace the AZO bottom layer with V_2_O_5_, to develop an all-in-one selective contact plus a transparent electrode for silicon-based solar cells. The V_2_O_5_ layer is needed to act as a dopant-free hole-selective layer. Even if they have clearly different electronic properties, replacing AZO by V_2_O_5_ as the bottom layer does not change much the transparency of the stack. The transfer-matrix method (TMM) algorithm developed in our previous work was used to predict the behavior of multilayer thin film structures [23]. The TMM algorithm for V_2_O_5_/Ag:Al/AZO multilayers predicted the optical performance of these structures on c-Si and, by integrating the air-mass 1.5 global irradiance (AM 1.5), the total photogenerated current J_ph_ was calculated. Figure 9 shows the expected mapping of the current density for varying thickness of V_2_O_5_ and AZO with a fixed thickness of Ag:Al intermediate layer of 7.1 nm. From the simulation the optimum thickness for the finest optical properties and high photogenerated current for the AZO layer was between 40 and 80 nm. As far as the optimum thickness for V_2_O_5_ layer is concerned, the best performance could be expected below 20 nm or above 70 nm. However, we have some additional constraints for the implementation in the solar cell device. First, the thickness of the V_2_O_5_ layer should not be less than 20 nm to prevent damage at the interface during the sputtering step. On the other hand, it cannot be too thick, such as 100 nm, because of its insulating nature. The same calculation considering the whole AZO/Ag:Al/AZO stack deposited on the V_2_O_5_ selective layer predicted a reduction in the photogenerated current of about 2–3%. Thus, a direct V_2_O_5_/Ag:Al/AZO stack on silicon was preferred for device testing. The solar cells were fabricated with a fixed thickness of AZO layer at 50 nm, and different thicknesses of V_2_O_5_ layer from 20 to 50 nm.

As for the primary study the above conclusions were used, four different solar cell architectures (i.e., N1, N2, N3 and N4) were fabricated using different thicknesses for the V_2_O_5_ layer (25 nm, 35 nm, 50 nm and 50 nm respectively) as the hole-selective contact. Out of these, for N1, N2 and N3 cells V_2_O_5_/Ag:Al/AZO multilayer acted as hole transport and transparent conducting layer, while in N4 cell structure it was AZO/ V_2_O_5_ multilayer, i.e., without the metallic layer Ag:Al. For these studies the thickness of the AZO and Ag:Al layer was kept constant at 50 nm and 7.1 nm respectively. The thickness of the layers was chosen from the electrical, optical, and topographical aforementioned studies on the films of AZO and Ag:Al.

**Figure 10:**
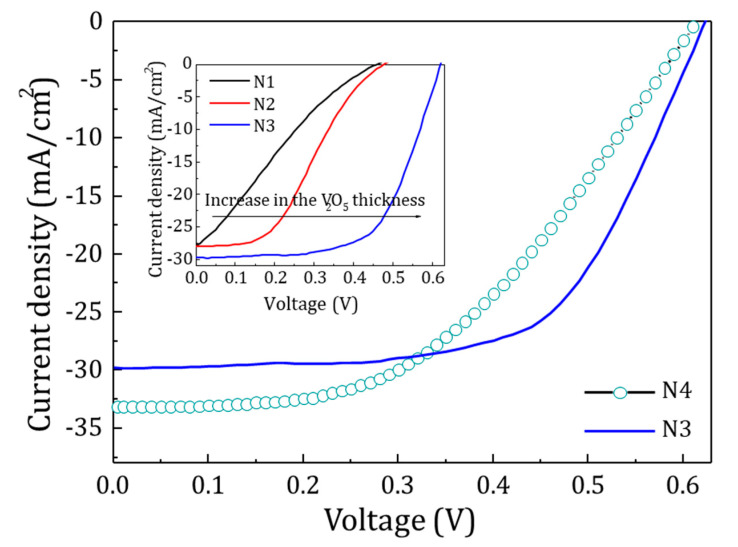
J-V curve measured under AM 1.5 irradiance (100 mW/cm^2^). N1, N2, N3 represents the devices with thickness of the V_2_O_5_ layer as 25 nm, 35 nm, 50 nm and N4 without the metallic layer. Higher V_oc_ of 631 mV for device structure N3 confirms the high capability of AZO/Ag:Al/V_2_O_5_ structures over the other transparent electrodes.

The J-V curves of devices N1, N2 and N3 showed that as the thickness of the V_2_O_5_ layer increased, there was a huge increase in the V_oc_ from 459 mV to 631 mV and in the fill factor (FF) from 22 to 61% (inset of Figure 10). This variation could be explained by the formation of defects in the V_2_O_5_ layer during sputtering because of Ag and Al ion bombardment. As the thickness of the V_2_O_5_ layer increases the damaging effect decreases and the inversion layer made by V_2_O_5_ becomes sufficient to improve the V_oc_ and other parameters of the cell. N4 device was fabricated with 50 nm thickness of the V_2_O_5_ layer, hence reducing the bombardment defects and improving the optical parameters. This device without the Ag:Al metallic layer shows a V_oc_ = 614 mV, J_sc_ = 33.1 mA, FF = 47.5% and an efficiency η = 9.6% (Figure 10). The device N3 with same thickness of V_2_O_5_, shows V_oc_ = 631 mV, J_sc_ = 29.8 mA, FF = 61.2% and η = 11.5%. These increases in V_oc_, FF and η in the device N3 could be directly attributed to the presence of the metallic Ag:Al layer which decreased the sheet resistance. All devices were measured under a simulated AM 1.5 irradiance of 100 mW/cm^2^. The photovoltaic parameters of all the cells are given in Table 2.

**Figure 11:**
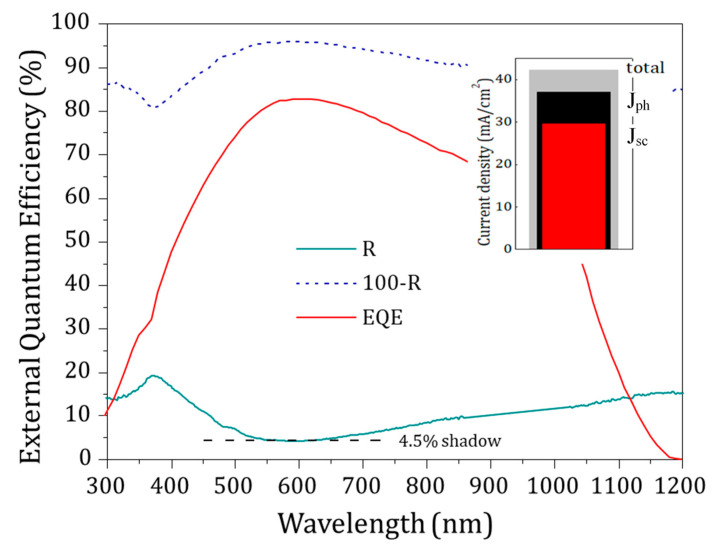
EQE curve of the solar cell together with its front reflectance spectra. Inset shows charge-carrier generation and different loss mechanisms balanced for an AM 1.5 irradiance.

External quantum efficiency (EQE) of the solar cell with its reflectance spectrum are plotted in Figure 11. The EQE dropped at long wavelengths photon energies lower than the Si bandgap (λ > 1100 nm). On the other hand, at short wavelengths (λ < 500 nm) the EQE was reduced because of the absorption in the front layers (DMD) and recombination at the front interface. A balance of the current entities calculated by integrating the solar spectrum is computed in the inset, taking into account optical and internal recombination losses.

The maximum photocurrent that could be obtained for photon energies higher than the Si bandgap of 1.1 eV was 42.3 mA/cm^2^. Reflectance losses of 11.3% on the front surface of the cell reduced the photogenerated current to 37.5 mA/cm^2^. The final photogenerated current value limits to 29.8 mA/cm^2^ due to the internal loss of 20.6% in the solar cell. Photocurrent losses in the c-Si bulk and the rear contact were expected to be very low accounting for the high-quality substrate and rear surface passivated contact. Thus, the 20.6% internal photocurrent loss could be explained by the parasitic absorption in the vanadium oxide layer and mainly to the absorption in the Ag:Al metallic layer. The degradation of V_2_O_5_ during sputtering also increased the internal recombination that eventually led to a reduction in the J_sc_ value. Further optimization in the thickness of the vanadium oxide layer in the V_2_O_5_/Ag:Al/AZO stack could reduce the damage during the sputtering. The fabrication of solar cells on textured wafers assuming the same internal losses could also increase the J_sc_ value up to 2–3 mA/cm^2^. Finally, controlling the thickness of Ag:Al and AZO layers could lead to further improvement in the photogenerated J_sc_ value.

## 4. Conclusions

An all-in-one excellent transparent conductor and hole-selective contact layer V_2_O_5_/Ag:Al/AZO was developed. This substitutes for traditional TCOs and henceforth reduces the need for more expensive standard ITO electrodes. Moreover, the deposition employed magnetron sputtering method, which is more industrial than the thermal evaporation technique. At first, the successful TCO substitute was achieved through the fabrication and optimization of ultra-thin Ag:Al layer as an intermediate layer for the AZO-based DMD structure AZO/Ag:Al/AZO. Al doping in Ag layer helped to obtain ultra-thin smooth metallic films of higher transmittance and lower sheet resistance. The optimized AZO/Ag:Al/AZO multilayer structure showed a sheet resistance of 48 Ω/Sq and 80% transmittance in the visible region of the spectra.

Then, in order to develop a hole-selective layer for the fabrication of solar cells the above TCO multi-structure was reconfigured, i.e., by replacing the bottom AZO layer by V_2_O_5_. Hence forming a dopant-free V_2_O_5_/Ag:Al/AZO all-in-one TCO and hole-selective contact. Afterwards, solar cells were fabricated using this multilayer with varied thicknesses of the bottom V_2_O_5_ layer. The solar cell on n-type c-Si with 50 nm thickness of V_2_O_5_ yielded a PCE of 11.5% with remarkably high V_oc_ of 631 mV and J_sc_ of 29.8 mA/cm^2^ showing its potential as an excellent TCO and as a hole-selective contact. The lower fill factor of 61.2% points out room for improvement in the contact by further optimization in the thickness of V_2_O_5_. Although we analyzed the potential of these structures on heterojunction solar cells, the concept could be applied to many other optoelectronic devices.

## Figures and Tables

**Table 1:** Comparison of FOM values for thin films and the multi-layer structure fabricated. AZO/Ag:Al/AZO structures at room temperature show the highest FOM factor, which indicates the potential for transparent conducting oxide.

	Temperature (°C)	FOM (10^−3^Ω^−1^)
AZO *	RT	0.02
AZO ^#^	RT	0.35
AZO ^#^	100	1.4
AZO^#^/Ag:Al *^#^/AZO^#^	RT	1.9
ITO *	100	1.1

* Deposited in Ar atmosphere; ^#^ Deposited in Ar:H_2_ atmosphere.

**Table 2:** The photovoltaic parameters (Open-circuit voltage V_oc_, short-circuit current density J_sc_, Fill factor FF, Photovoltaic conversion efficiency η) of the solar cells fabricated. N1, N2, N3 are the cells with V_2_O_5_ layer thicknesses 25 nm, 35 nm, 50 nm respectively. N4 is the cell without the Ag:Al metallic layer with 50 nm thickness of V_2_O_5_ layer. The increase in the V_oc_ and FF of the cells could be observed as the thickness of the V_2_O_5_ increases. A huge decrease in the FF is observed in N4 due to the high sheet resistance of the layers.

	N1	N2	N3	N4
V_oc_ (mV)	459 ± 1	479 ± 1	631 ± 1	614 ± 1
J_sc_ (mA/cm^2^)	28.0 ± 0.2	28.1 ± 0.2	29.8 ± 0.2	33.1 ± 0.2
FF (%)	17.5 ± 0.5	38.6 ± 0.5	61.2 ± 0.5	47.5 ± 0.5
η (%)	2.2 ± 0.2	5.2 ± 0.2	11.5 ± 0.2	9.6 ± 0.2
